# The association between prenatal famine, DNA methylation and mental disorders: a systematic review and meta-analysis

**DOI:** 10.1186/s13148-023-01557-y

**Published:** 2023-09-16

**Authors:** Heike Eichenauer, Ulrike Ehlert

**Affiliations:** Clinical Psychology and Psychotherapy, University of Zurich, Binzmühlestrasse 14, 8050 Zurich, Switzerland

**Keywords:** DNA methylation, Mental disorders, Prenatal famine exposure, Epigenetic, Pregnancy

## Abstract

**Background:**

Undernutrition in pregnant women is an unfavorable environmental condition that can affect the intrauterine development via epigenetic mechanisms and thus have long-lasting detrimental consequences for the mental health of the offspring later in life. One epigenetic mechanism that has been associated with mental disorders and undernutrition is alterations in DNA methylation. The effect of prenatal undernutrition on the mental health of adult offspring can be analyzed through quasi-experimental studies such as famine studies. The present systematic review and meta-analysis aims to analyze the association between prenatal famine exposure, DNA methylation, and mental disorders in adult offspring. We further investigate whether altered DNA methylation as a result of prenatal famine exposure is prospectively linked to mental disorders.

**Methods:**

We conducted a systematic search of the databases PubMed and PsycINFO to identify relevant records up to September 2022 on offspring whose mothers experienced famine directly before and/or during pregnancy, examining the impact of prenatal famine exposure on the offspring’s DNA methylation and/or mental disorders or symptoms.

**Results:**

The systematic review showed that adults who were prenatally exposed to famine had an increased risk of schizophrenia and depression. Several studies reported an association between prenatal famine exposure and hyper- or hypomethylation of specific genes. The largest number of studies reported differences in DNA methylation of the *IGF2* gene. Altered DNA methylation of the *DUSP22* gene mediated the association between prenatal famine exposure and schizophrenia in adult offspring. Meta-analysis confirmed the increased risk of schizophrenia following prenatal famine exposure. For DNA methylation, meta-analysis was not suitable due to different microarrays/data processing approaches and/or unavailable data.

**Conclusion:**

Prenatal famine exposure is associated with an increased risk of mental disorders and DNA methylation changes. The findings suggest that changes in DNA methylation of genes involved in neuronal, neuroendocrine, and immune processes may be a mechanism that promotes the development of mental disorders such as schizophrenia and depression in adult offspring. Such findings are crucial given that undernutrition has risen worldwide, increasing the risk of famine and thus also of negative effects on mental health.

**Supplementary Information:**

The online version contains supplementary material available at 10.1186/s13148-023-01557-y.

## Background

Unfavorable environmental conditions during pregnancy have been shown to promote the onset of mental disorders in the offspring [1–3] via epigenetic mechanisms [4–6]. One epigenetic mechanism that can be changed by adverse intrauterine exposure and influences the development of offspring health is deoxyribonucleic acid (DNA) methylation [5, 7–10]. DNA methylation is the addition of methyl groups to cytosine-guanine dinucleotides (CpG), with the potential to regulate gene expression [11–15]. For instance, Palma-Gudiel et al. [16] reported increased methylation of the glucocorticoid receptor gene (*NR3C1*), a gene involved in the regulation of the hypothalamic–pituitary–adrenal (HPA) axis in the offspring, following exposure to prenatal stress. Increased *NR3C1* methylation has, in turn, been associated with mental disorders [17–19] such as depression [20].

Undernutrition in pregnant women is an unfavorable environmental condition that can affect the intrauterine development and may thus have long-lasting detrimental consequences for the mental health of the offspring later in life [21]. The effect of prenatal undernutrition on mental health can be analyzed through natural experiments (quasi-experimental studies), in which undernutrition (e.g. famine) occurs naturally in a specific population [22, 23]. Meta-analytic results have already demonstrated an increased risk of suffering from psychotic, affective, and personality disorders in adults who were exposed to famine during prenatal development [24].

One important mechanism to explain how unfavorable maternal food consumption leads to an increased susceptibility to mental disorders in the offspring in adulthood may be altered DNA methylation patterns [25–27]. Rijlaarsdam et al. [28] reported that an unhealthy high-fat and high-sugar prenatal diet was positively associated with changes in the insulin-like growth factor gene (*IGF2*) in the offspring, which was in turn related to increased attention deficit hyperactivity disorder (ADHD) symptoms in adolescence [28]. Moreover, hypomethylation of this *IGF2* gene has been found in adult offspring who were prenatally exposed to famine [29]. Less is known, however, about whether altered DNA methylation mediates the effects of prenatal famine exposure on mental disorders in the offspring.

In summary, undernutrition during pregnancy appears to increase the susceptibility to mental disorders in the offspring. However, the aforementioned meta-analysis did not include a quality assessment [24]. To date, therefore, no quality assessment has been conducted on the myriad of published studies examining the effects of prenatal famine exposure on offspring mental health. Moreover, it remains to be elucidated whether changes in DNA methylation are the mechanism linking prenatal famine exposure to the development of mental disorders in adult offspring. The purpose of this study is thus to provide the first systematic review of the existing literature on the impact of prenatal famine exposure on offspring mental health and altered DNA methylation, and to integrate the findings by means of a meta-analysis.

## Methods

### Search strategy

We conducted a literature search of the databases PubMed and PsycINFO to identify relevant records up to September 2022. The search strategies included the words (a) “famine” and related terms, (b) “pregnancy” and related terms, (c) “DNA methylation” and related terms, or (d) “mental disorders” and related terms. The search followed a systematic approach in accordance with the Preferred Reporting Items for Systematic review and Meta-Analysis Protocols (PRISMA-P) guidelines [30]. This systematic review and meta-analysis was registered on the Open Science Framework (OSF): osf.io/3hn5p.

### Screening and selection procedure

First, duplicates of the identified records were removed. Titles and abstracts were screened, and records that did not meet the eligibility criteria, such as non-human studies and non-empirical research, were excluded. The articles yielded by the literature search were screened and selected using the following inclusion criteria: (1) offspring whose mothers experienced famine during pregnancy and including either (2) a measure of DNA methylation or (3) a measure of psychopathology. A full-text reading of all remaining articles was performed. Studies were included in the meta-analyses if they (1) used the same questionnaire to measure symptoms of psychopathology, (2) included a categorical outcome (mental disorders) irrespective of which clinical interview was used to establish the diagnosis, and (3) provided adequate data for statistical analysis.

### Data extraction

Included articles were examined for information about the first author, year of publication, cohort, sample description, assessment of symptoms of psychopathology, and main results. Articles on DNA methylation were examined for information about chromosome number and location, gene, number of CpGs, method for DNA methylation analysis, and main results. Data extraction was performed by one of the authors (HE) and a research assistant. Risk of bias was assessed using a modified version of the Newcastle–Ottawa scale [31, 32], containing the following seven items: sampling representativeness, sample size, exposure definition, famine severity assessment, confounding adjustment, outcome assessment, and statistical methods. Each item was scored as either good, fair, or poor [31]. The items outcome assessment and sample size were modified for studies on mental disorders, epigenome-wide DNA methylation analyses, and targeted candidate gene analyses (see Additional file 1: Tables S1–S3). Risk of bias assessment was performed by one of the authors (HE) and a senior researcher from our workgroup.

### Data analysis

To assess the association between prenatal famine exposure and symptoms of psychopathology or mental disorders in adulthood, we calculated the effect size across studies as the overall pooled log10 odds ratio (logOR) of the number of individuals with and without symptoms or a mental disorder in the prenatal famine group and in the control group. The logOR was used for the depression and schizophrenia studies. The control group consisted of offspring who were exposed to famine during childhood (non-prenatal famine exposure) and/or offspring who were not exposed to famine at all (non-exposure). For two studies that used the Hospital Anxiety and Depression Scale (HADS), we used means and standard deviations to calculate Hedges’ g. One of these studies did not report the specific standard deviations for each of the two subscales of the HADS (anxiety and depression) and instead only provided overall standard deviations, which were therefore used as a reference. Results were considered statistically significant if the *p* value was < 0.05. Meta-analyses were conducted if at least two studies used the same outcome measurement. Studies with insufficient data were only included in the systematic review, and not in the meta-analyses. Random-effects meta-analyses were conducted using the meta-analysis function integrated in SPSS version 28.0.1.1, which also allowed us to create forest plots. The Q and I^2^ statistics were calculated to assess the heterogeneity of the included studies. Subgroup analyses were performed to detect whether a more homogenous effect size could be calculated. Following the Cochrane Handbook for Systematic Reviews of Interventions [33], when 10 or more studies were included in our meta-analyses, we used the trim-and-fill procedure and visual inspection of funnel plots to detect publication bias [34].

## Results

### Search results

**Fig. 1:**
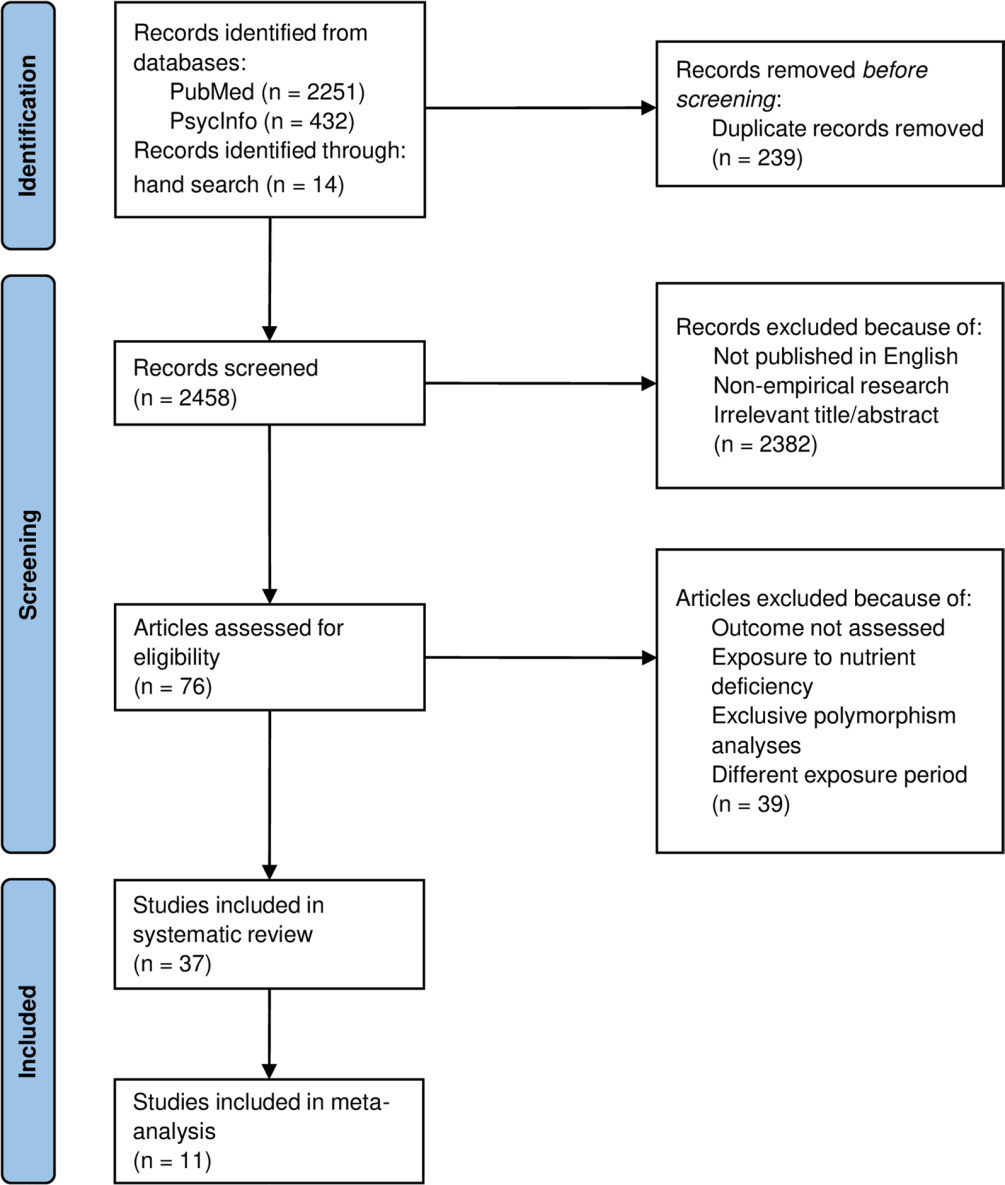
Screening and selection process of studies displayed by a PRISMA flowchart

The literature search yielded 2697 articles, of which 239 were duplicates and removed. Of the remaining 2458 articles, a further 2382 were excluded due to publication in a language other than English, non-empirical research, or irrelevant title/abstract. Of the final 76 articles assessed for eligibility, 39 were excluded for as they did not assess the outcome, only examined exposure to nutrient deficiency, were exclusively polymorphism analyses, or assessed different exposure periods. Thus, in total, 37 studies were eligible for data extraction and were included in this systematic review. Of these studies, 22 reported effects of prenatal famine exposure on symptoms of psychopathology or mental disorders, and 14 studies reported effects of famine during pregnancy on DNA methylation. The remaining study analyzed the mediating effect of DNA methylation on mental disorders in adults prenatally exposed to famine. Eleven of the 37 studies reported sufficient data to be included in meta-analyses. The study selection is summarized in Fig. 1.

### Study characteristics

Characteristics of the included studies are shown in Tables 1, 2, 3 and 4. Articles were published between 1992 and 2022. All participants were adults. The sample size ranged from 13 to 494,684. All studies focused either on the Dutch Famine (1944–1945) or the Chinese Famine (1959–1961), with one exception, the Bangladesh Famine (1974–1975). Individuals without prenatal famine exposure were either born after the famine (*non-exposure*: had not experienced famine in their life) or before the famine (*non-prenatal exposure*: experienced famine during infancy, childhood, adolescence, or adulthood). Most DNA methylation studies (67%) used either sibling or time controls. Sibling controls were siblings of prenatally exposed adults and were mostly younger than their exposed siblings. Time controls were adults who were born either before or after the famine. As the respective authors did not specify how many control adults were in each group, it was not possible to assign them to the non-prenatal exposure or non-exposure group. Periconceptional exposure referred to exposure to famine during conception and the 1st trimester.

**Table 1:** Effects of prenatal exposure to famine on mental disorders/symptoms in offspring

References	Cohort	Sample description of groups with prenatal, non-prenatal or non-exposure	Assessment of symptoms of psychopathology	Main results
Zhou et al. [42]	Chinese Famine*^a^	Prenatal exposure N = 1575, ♀ not stated, mean age 50	CES-D	Increased depressive symptoms after prenatal exposure and non-prenatal exposure compared to non-exposure***
		Non-prenatal exposure N = 9138, ♀ not stated, age 57–69		
		Non-exposure N = 1968, ♀ not stated, mean age 47		
He et al. [43]	Chinese Famine*^a^	Prenatal exposure N = 76, ♀ = 48, mean age not stated	GDS	Increased risk of depression after prenatal exposure compared to non-prenatal exposure*
		Non-prenatal exposure N = 80, ♀ = 28, mean age not stated		
Franzek et al. [57]	Dutch Famine*^b^	Prenatal exposure N = 5549	Case records of individuals with addictive behaviors in the database of the Dutch mental health care organizations	Increased risk of addictive behaviors after prenatal exposure during 1st trim (in men)*** and 3rd trim (in women)*** compared to non-exposure
		1st trim = 1738, ♀ = 812		
		2nd trim = 568, ♀ = 287		
		3rd trim = 3243, ♀ = 1608		
		Non-exposure N = 11,630, ♀ = 5676		
		Age not stated		
van den Broek et al. [38]	Dutch Famine*^b^	Prenatal exposure N = 23, ♀ = 11	MHI-5	Poorer mental health after prenatal exposure compared to non-prenatal exposure** and non-exposure*
		Non-prenatal exposure N = 41, ♀ = 19		
		Non-exposure N = 83, ♀ = 34		
		Mean age of entire sample 57		
Franke et al. [45]	Dutch Famine*^b^	Prenatal exposure N = 41, ♀ = 22, mean age 67	HADS-A/-D	No significant differences between prenatal exposure, non-prenatal exposure and non-exposure in anxiety and depressive symptoms
		Non-prenatal exposure N = 35, ♀ = 21, mean age 69		
		Non-exposure N = 42, ♀ = 23, mean age 67		
He et al. [50]	Chinese Famine*^a^	Rural population N = 239,055, ♀ = 119,217, mean age not stated	Diagnosis of schizophrenia with ICD-10 semi-structured symptom checklist for mental disorders	Only in rural population, increased risk of schizophrenia after prenatal exposure compared to non-exposure*
		Urban population N = 148,038, ♀ = 73,471, mean age not stated		
		N for prenatal exposure, non-prenatal exposure and non-exposure not stated		
Li et al. [40]	Chinese Famine*^a^	Prenatal exposure N = 996	CES-D	More depressive symptoms after prenatal exposure during 1st and 2nd trim compared to non-exposure*
		Non-exposure N = 356		
		Trim and ♀ not stated		
		Age of entire sample > 45		
Li et al. [39]	Chinese Famine*^a^	Prenatal exposure N = 1847, ♀ = 1019	CES-D	Only in women, more depressive symptoms after prenatal exposure compared to non-exposure (significant, but *p* not stated)
		Non-exposure N = 2698, ♀ = 1671		
		Age of entire sample > 45		
Wang et al. [47]	Chinese Famine*^a^	Prenatal exposure N = 81,279, ♀ = 40,509	Diagnosis of schizophrenia with ICD-10 semi-structured symptom checklist for mental disorders	Increased risk of schizophrenia after prenatal exposure compared to non-prenatal exposure*** and non-exposure***
		Non-prenatal exposure N = 120,287, ♀ = 59,650		
		Non-exposure N = 150,429, ♀ = 75,470		
		Age not stated		
Huang et al. [46]	Chinese Famine*^a^	Prenatal exposure N = 1477, ♀ = 752	GHQ-12 and the presence (yes/no) of eight additional risk factors for mental disorders	In women, increased GHQ-12 scores** and risk of mental disorders** and in men, decreased GHQ-12 scores** after prenatal exposure compared to non-exposure
		Non-exposure N = 1029, ♀ = 514		
		Age not stated		
de Rooij et al. [44]	Dutch Famine*^b^	Prenatal exposure N = 334	HADS-A/-D	Only in men, higher HADS-D and HADS-A scores after prenatal exposure during 1st trim compared to non-prenatal exposure* and non-exposure*
		1st trim = 75, ♀ = 44, mean age 58		
		2nd trim = 121, ♀ = 76, mean age 58		
		3rd trim = 138, ♀ = 77, mean age 59		
		Non-prenatal exposure N = 253, ♀ = 136, mean age 59		
		Non-exposure N = 232, ♀ = 117, mean age 57		
Song et al. [51]	Chinese Famine*^a^	Prenatal exposure N = 81,318, ♀ = 40,415	Diagnosis of schizophrenia based on CCMD with a semi- structured interview	Increased risk of developing schizophrenia after non-exposure compared to prenatal exposure*
		Non-prenatal exposure N = 102,068, ♀ = 50,422		
		Non-exposure N = 110,970, ♀ = 56,706		
		Age of entire sample 22–32		
Stein et al. [41]	Dutch Famine*^b^	Prenatal exposure N = 411, mean age 59	CES-D	Increased depressive symptoms after periconceptional and prenatal exposure compared to time and sibling controls (significant, but *p* not stated)
		Periconceptional exposure N = 91		
		Time controls N = 218, mean age 59		
		Sibling controls N = 294, mean age 57		
		♀ not stated		
Xu et al. [36]	Chinese Famine*^a^	Prenatal exposure N = 126,579	Case records (1971–2001) of schizophrenia patients from Longquanshan hospital	Increased risk of schizophrenia after prenatal exposure compared to non-prenatal exposure*** and non-exposure***
		Non-prenatal exposure N = 329,189		
		Non-exposure N = 494,684		
		Age and ♀ not stated		
Franzek et al. [56]	Dutch Famine*^b^	Prenatal exposure N = 2202, ♀ = 1055	Case records of addictive disorder patients in the database of the Dutch mental health care organization	Increased risk of addictive disorders, especially in men*, after prenatal exposure during 1st trim compared to non-exposure**
		Non-exposure N = 5441, ♀ = 2753		
		Age not stated		
St. Clair et al. [35]	Chinese Famine*^a^	Prenatal exposure N = 141,713	Case records (1971–2001) of schizophrenia patients from Fourth People’s hospital	Increased risk of schizophrenia after prenatal exposure compared to non-prenatal exposure*** and non-exposure***
		Non-prenatal exposure N = 176,335		
		Non-exposure N = 243,647		
		Age and ♀ not stated		
Brown et al. [52]^c^	Dutch Famine*^b^	Prenatal exposure N = 41,969	Case records of patients with major affective disorder from the Dutch national psychiatric registry from 1970 to 1996	Increased risk of major affective disorder requiring hospitalization after prenatal exposure during 2nd***and 3rd trim** for men and during 3rd trim* for women compared to non-exposure
		1st trim = 9656, ♀ = 4672		
		2nd trim = 14,645, ♀ = 7185		
		3rd trim = 17,668, ♀ = 8727		
		Non-exposure N = 115,877, ♀ = 56,472		
		Age of entire sample ≥ 18		
Neugebauer et al. [54]	Dutch Famine*^b^	Severe prenatal exposure N = 14,310	Non-standardized diagnosis of ASPD in men at time of medical examination for military induction	Increased risk of ASPD after severe prenatal exposure during 1st and/ or 2nd trim compared to non-prenatal and non-exposure (significant, but *p* not stated)
		1st and/or 2nd trim = 9252, 3rd trim = 5058		
		Non-prenatal and non-exposure N = 45,007		
		Age of entire sample ≥ 18, ♀ not stated		
Hoek et al. [55]	Dutch Famine*^b^	Prenatal exposure (Aug-Oct 1945) N = 2610	Diagnosis of schizoid personality disorder in men with ICD-6 and ICD-9	Increased risk of schizoid personality disorder after prenatal exposure (Oct-Dec) compared to non-prenatal and non-exposure*
		Prenatal exposure (Oct-Dec 1945) N = 2056		
		Non-prenatal and non-exposure N = 64,265		
		Age of entire sample > 18, ♀ not stated		
Susser et al. [48]^c^	Dutch Famine*^b^	Conception at peak N = 4190, ♀ = 2006	Case records of patients with schizophrenia from the Dutch national psychiatric registry from 1970 to 1992	Only for conception at peak of famine, increased risk of schizophrenia compared to non-prenatal and non-exposure**
		Conception not at peak N = 5466, ♀ = 2666		
		Non-prenatal and non-exposure N = 136,691, ♀ = 66,748		
		Age of entire sample 24–48		
Brown et al. [53]^c^	Dutch Famine*^b^	Prenatal exposure N = 41,969	Case records of patients with major affective disorders from the Dutch national psychiatric registry from 1978 to 1991	Only in men, increased risk of major affective disorders after prenatal exposure during 2nd trim compared to non-prenatal and non-exposure*
		1st trim = 9656, ♀ = 4672		
		2nd trim = 14,645, ♀ = 7185		
		3rd trim = 17,668, ♀ = 8727		
		Non-prenatal and non-exposure N = 397,052		
		1st trim = 136,691, ♀ = 66,748		
		2nd trim = 131,702, ♀ = 64,235		
		3rd trim = 128,659, ♀ = 62,693		
		Age of entire sample 32–47		
Susser et al. [49]^c^	Dutch Famine*^b^	Prenatal exposure 1st trim = 9656, ♀ = 4672	Case records of patients with schizophrenia from the Dutch national psychiatric registry from 1978 to 1989	Only in women, increased risk of schizophrenia after prenatal exposure during 1st trim compared to non-prenatal and non-exposure (significant, but *p* not stated)
		Non-prenatal and non-exposure N = 116,934, ♀ = 57,034		
		Age of entire sample ≥ 19		

*^a^Chinese Famine:1959–1961; *^b^Dutch Famine:1944–1945; ^c^possible sample overlap between [48, 49] as well as [52, 53]; *ASPD* Antisocial Personality Disorder, *CCMD* Chinese Classification of Mental Disorders, *CES-D* Center for Epidemiologic Studies Depression Scale, *GDS* Geriatric Depression Scale, *GHQ -12* General Health Questionnaire, *HADS-A/-D* Hospital Anxiety and Depression Scale, *ICD* International Statistical Classification of Diseases and Related Health Problems, *MHI-5* Mental Health Inventory, *trim* Trimester; *p ≤ 0.05, **p ≤ 0.01, ***p ≤ 0.001

**Table 2:** Effects of prenatal exposure to famine on (epi)genome-wide DNA methylation of the offspring

References	Cohort	Sample description of groups with prenatal, non-prenatal or non-exposure	Chromosome	Gene	No. CpG	DNA methylation analysis from blood	Main results
Li et al. [65]	Chinese Famine*^a^	Prenatal exposure N = 79, ♀ = 49, mean age 57	–	–	–	Illumina Infinium Methylation EPIC BeadChip	No significant differences in DNA methylation (DMRs and CpGs) between prenatal exposure and non-exposure after controlling for multiple testing
		Non-exposure N = 105, ♀ = 31, mean age 53					
Jiang et al. [63]	Chinese Famine*^a^	Prenatal exposure N = 46, ♀ = 24, mean age 52	–	–	–	Illumina Infinium Human Methylation 850 K BeadChip	601 DMRs with significant* hypermethylation and 360 DMRs with significant* hypomethylation after prenatal exposure compared to time controls (for more details, see Fig. 1 in [63])
		Time controls N = 46, ♀ = 24, mean age 53					
He et al. [58]	Chinese Famine*^a^	Early prenatal exposure N = 25, ♀ = 15, mean age 50	chr3:48481268–48481793	*CCDC51/TMA7*	13	Illumina Infinium Human Methylation 450 BeadChip	613 DMRs with significant methylation (significance and direction of effect not stated): specifically, hypomethylation of CCDC51/TMA7***, ENO2*** and ZNF226** after early prenatal exposure compared to non-exposure
		Non-exposure N = 54, ♀ = 33, mean age 47	chr12:7023752–7024121	*ENO2*	4		
			chr19:44669146–44669354	*ZNF226*	5		
Tobi et al. [64]^d^	Dutch Famine*^b^	Prenatal exposure N = 348, ♀ = 188, mean age 59	chr21:43655316	*ABCG1*	1	Illumina Infinium Human Methylation 450 BeadChip	Of 342,596 CpGs, 17 CpGs with significant differences in methylation between prenatal exposure and sibling controls: hypermethylation of ABCG1**, CCDC155***, FAM150B***, METTL8*, PNPO***, PPAP2C***, SLC38A2***, SYNGR1**, TACC1*** and ZNF385A*** and hypomethylation of CRELD2**, LRRC8D***, LOC100132354***, OSBPL5/MRGPRG***, TXNIP** and PFKFB3*
			chr19:49891270	*CCDC155*	1		
		Sibling controls N = 463, ♀ = 264, mean age 58	chr19:49891574	*CCDC155*	1		
			chr22:50327986	*CRELD2*	1		
			chr2:366113	*FAM150B*	1		
			chr6:43894639	*LOC100132354*	1		
			chr1:90288099	*LRRC8D*	1		
			chr2:172203847	*METTL8*	1		
			chr11:3225076	OSBPL5/MRGPRG	1		
			chr10:6214026	*PFKFB3*	1		
			chr17:46022809	*PNPO*	1		
			chr19:292167	*PPAP2C*	1		
			chr12:46737123	*SLC38A2*	1		
			chr22:39759864	*SYNGR1*	1		
			chr1:145441552	*TXNIP*	1		
			chr8:38586183	*TACC1*	1		
			chr12:54764265	*ZNF385A*	1		
Finer et al. [59]	Bangladesh Famine*^c^	Prenatal exposure N = 40 Non-prenatal exposure N = 49 Non-exposure N = 54	–	–	–	Illumina Infinium Human Methylation 450 BeadChip	No significant differences in DNA methylation between prenatal exposure, non-prenatal exposure and non-exposure
Tobi et al. [60]^d^	Dutch Famine*^b^	Prenatal exposure during any week N = 348, ♀ = 188, mean age 59; conception N = 74, ♀ not stated; weeks 1–10 = 73, ♀ = 39; weeks 11–20 = 123, ♀ = 66; weeks 21–30 = 143, ♀ = 72; weeks 31-delivery = 128, ♀ = 66	chr2:366113	*FAM150B/TMEM18*	1	Illumina Infinium Human Methylation 450 BeadChip	Hypomethylation of TMEM105/SLC38A10* after exposure during conception compared to time and sibling controls; hypermethylation of FAM150B/TMEM18**, PPAP2C*, SLC38A2** and hypomethylation of OSBPL5/ MRGPRG* after exposure during weeks 1–10 compared to time and sibling controls; hypermethylation of ZNF385A* and TACC1* after exposure during any week compared to time and sibling controls
			chr11:3225076	*OSBPL5/MRGPRG*	1		
			chr19:292167	*PPAP2C*	1		
			chr12:46737123	*SLC38A2*	1		
			chr8:38586183	*TACC1*	1		
			chr17:79283915	*TMEM105/SLC38A10*	1		
			chr12:54764265	*ZNF385A*	1		
		Time controls N = 160, ♀ = 88, mean age 59					
		Sibling controls N = 303, ♀ = 176, mean age 57					
Tobi et al. [61]	Dutch Famine*^b^	Periconceptional exposure N = 24, ♀ = 12, mean age 58	–	–	–	RRBS	181 DMRs with 60.8% significantly hypermethylated and 39.2% hypomethylated after periconceptional exposure compared to sibling controls (for more details and significance see S1 in [61])
		Sibling controls N = 24, ♀ = 12, mean age 57					
Tobi et al. [66]	Dutch Famine*^b^	Periconceptional exposure N = 60, ♀ = 32, mean age 58	–	*LINE-1*^e^	–	Pyrosequencing	No significant difference in global DNA methylation between periconceptional exposure and sibling controls
		Sibling controls N = 60, ♀ = 32, mean age 57					
Lumey et al. [62]	Dutch Famine*^b^	Prenatal exposure N = 350, ♀ = 189, mean age 59	chr17	*Sat2*	–	MethyLight	No significant differences in global DNA methylation between prenatal exposure and time and sibling controls
		Time controls N = 290, ♀ = 154, mean age 59	–	*LINE-1*^e^		Pyrosequencing	
		Sibling controls N = 307, ♀ = 175, mean age 57				LUMA	

*^a^Chinese Famine: 1959–1961, *^b^Dutch Famine: 1944–1945; *^c^Bangladesh Famine: 1974–1975, ^d^sample overlap between [60, 64], ^e^estimate of global methylation; *ABCG1* ATP Binding Cassette Subfamily G Member 1, *CCDC51* Coiled-Coil Domain Containing 51, *CCDC155* Coiled-Coil Domain Containing 155, *CRELD2* Cysteine Rich with EGF-Like Domains 2, *DMR* Differentially Methylated Region, *ENO2* Enolase 2, *FAM150B* Family with sequence similarity 150 member B*, LINE-1* Long interspersed nucleotide element-1, *LOC100132354* LOC100132354*, LRRC8D* Leucine Rich Repeat Containing 8 VRAC Subunit D, *LUMA* Luminometric methylation assay, *METTL8* Methyltransferase 8, *MRGPRG* MAS-related GPR family member G, *OSBPL5* Oxysterol binding protein-like 5, *PFKFB3* 6-Phosphofructo-2-Kinase/Fructose-2,6-Biphosphatase 3, *PNPO* Pyridoxamine 5′-Phosphate Oxidase, *PPAP2C* Phosphatidic acid phosphatase 2c, *RRBS* Reduced representation bisulfite sequencing, *Sat2* Satellite repeat-2, *SLC38A2* Solute carrier family 38 member 2, *SLC38A10* Solute Carrier Family 38 Member 10, *SYNGR1* Synaptogyrin 1, *TACC1* Transforming Acidic Coiled-Coil Containing Protein 1*, TMA7* Translation machinery-associated protein 7, *TMEM18* Transmembrane protein 18, *TMEM105* TMEM105 long non-coding RNA, *TXNIP* Thioredoxin Interacting Protein, *ZNF226* Zinc finger protein 226, *ZNF385A* Zinc finger protein 385A; *p ≤ 0.05, **p ≤ 0.01, ***p ≤ 0.001

**Table 3:** Effects of prenatal exposure to famine on targeted DNA methylation of the offspring

References	Cohort	Sample description of groups with prenatal, non-prenatal or non-exposure	Chromosome	Gene	No. CpG	DNA methylation analysis from blood	Main results
Jiang et al. [63]	Chinese Famine*^a^	Prenatal exposure N = 194, ♀ = 89, mean age 52	chr3:148416100–148416355	*AGTR1*	1	Bisulfite sequencing	Hypomethylation of *AGTR1* (cg13528513)*, *AGTR1 (*cg20906621)**, and PRKCA** after prenatal exposure compared to time controls
			chr3:148418205–148418530	*AGTR1*	1		
		Time controls N = 192, ♀ = 94, mean age 52	chr17:64649040–64649570	*PRKCA*	1		
Wang et al. [72]^d^	Chinese Famine*^a^	Prenatal exposure N = 75, ♀ = 38, mean age 55	chr11:2126035–2126372	*IGF2*	8	EpiTYPER	Hypermethylation of *IGF2* CpG2* and *INSR* CpG1**, 4**, 5** and 7** after prenatal exposure compared to time controls; no significant differences for other CpGs
			chr19:7110130–7110574	*INSR*	9		
		Time controls N = 160, ♀ = 80, mean age 55					
Wang et al. [74]^d^	Chinese Famine*^a^	Prenatal exposure N = 75, ♀ = 38, mean age 55	chr11:68286513–68286952	*CPT1A*	11	EpiTYPER	Hypermethylation of *INSR* CpG1***, 4***, 5** and 7*** after prenatal exposure compared to time controls; no significant differences for *CPT1A*
			chr19:7110130–7110574	*INSR*	9		
		Time controls N = 160, ♀ = 80, mean age 55					
Finer et al. [59]	Bangladesh Famine*^c^	Prenatal exposure N = 13	chr6:151646312–151647133	*AKAP12*	9	Bisulfite Pyrosequencing	Hypomethylation of VTRNA2-1* and EXD3* after prenatal exposure compared to non-prenatal exposure; hypermethylation of PAX8*** and hypomethylation of ZFP57*** and PRDM9*** after prenatal exposure compared to non-prenatal and non-exposure; no significant differences for other genes
		Non-prenatal exposure N = 30	chr12:57040045–57040204	*ATP5B*	3		
		Non-exposure N = 18	chr2:74357713–74357851	*BOLA*	2		
		Age and ♀ not stated	chr9:140311919–140311437	*EXD3*	3		
			chr6:32729442–32729847	*HLA-DQB2*	15		
			chr5:191242–192103	*LRRC14B*	11		
			chr18:77918588–77918142	PARD6G	4		
			chr2:113992762–113993313	*PAX8*	8		
			chr17:17109570–17110120	*PLD6*	8		
			chr5:23507030–23507752	*PRDM9*	7		
			chr4:155702411–155702351	*RBM46*	11		
			chr13:36944640–36944649	*SPG20*	2		
			chr5:135415762–135416613	*VTRNA2-1*	15		
			chr6:29648345–29649024	*ZFP57*	18		
			chr4:2366672–2367137	*ZFYVE28*	7		
			chr1:227746294–227746111	*ZNF678*	3		
Tobi et al. [61]^d^	Dutch Famine*^b^	Periconceptional exposure N = 60, ♀ = 32, mean age 58	chr10:73227653–73227914	*CDH23*	4	EpiTYPER	Hypermethylation of *CDH23* CpG1**, 2*, 3–4**, *CPT1A* CpG 8–10*, 12*, *INSR* CpG2**, *SMAD7* CpG1**, 2*, 3–4**, 5–7* and hypomethylation of *KLF13* CpG2*, 4- 7*,9* after periconceptional exposure compared to sibling controls; no significant differences for *RFTN1*
			chr11:68286598–68286810	*CPT1A*	8		
		Sibling controls N = 60, ♀ = 32, mean age 57	chr19:7110140–7110418	*INSR*	3		
			chr18:44677194–44677679	*SMAD7*	7		
			chr15:29425223–29425563	*KLF13*	6		
			chr3:16394247–16394578	*RFTN1*	11		
Tobi et al. [66]^d^	Dutch Famine*^b^	Periconceptional exposure N = 60, ♀ = 32, mean age 58	chr11:1975948–1976360	*H19 DMR*	9	EpiTYPER	Hypomethylation of *IGF2* DMR0 upstr. CpG1**, 2*, 3**, 4*, *IGF2* DMR0**, *IGF2* DMR0 downstr. CpG8*, 12–13***, *IGF2* DMR2 CTCF CpG1*,4*, INSIGF* and hypermethylation of *IGF2AS* DMR1 CpG41** and *IGF2AS* DMR1 CTCF CpG20**, 22* after periconceptional exposure compared to sibling controls; no significant differences for *IGF* DMR2 S.L. and* H19*
			chr11:2138912–2139216	*INSIGF*	n.s.		
		Sibling controls N = 60, ♀ = 32, mean age 57	chr11:2111300–2111791	*IGF2 DMR2* *S.L*	8		
			chr11:2112023–2112312	*IGF2 DMR2* *CTCF*	3		
			chr11:2117482–2117948	*IGF2AS*	12		
			chr11:2118126–2118422	*IGF2AS* *CTCF*	12		
			chr11:2125961–2126065	*IGF2 DMR0 upstr*	5		
			chr11:2126035–2126372	*IGF2 DMR0*	n.s.		
			chr11:2127117–2127220	*IGF2 DMR0 downstr*	3		
Veenendaal et al. [73]	Dutch Famine*^b^	Prenatal exposure N = 319	chr5:142782821–142783152	*GR 1-C*	n.s.	PCR	No significant differences for *GR1-C, LPL, PI3kinase* and *PPARγ* in each trimester compared to non-prenatal exposure and non-exposure
		1st trim = 73, ♀ = 42, mean age 58; 2nd trim = 112, ♀ = 68, mean age 58; 3rd trim = 134, ♀ = 75, mean age 59	chr8:19796366–19796515	*LPL*			
			chr5:67521933–67522282	*PI3kinase*			
			chr3:12392392–12392591	*PPARy*			
		Non-prenatal exposure N = 235, ♀ = 127, mean age 59					
		Non-exposure N = 205, ♀ = 103, mean age 57					
Tobi et al. [70]^d^	Dutch Famine*^b^	Group 1	chr9:106730323–106730642	*ABCA1*	22	EpiTYPER	Group 1
		Periconceptional exposure N = 60, ♀ = 32, mean age 58	chr19:50109726–50110115	*APOC*1	6		Hypermethylation of ABCA1*, IL-10***, LEP* and GNASAS*** and hypomethylation of INSIGF*** after periconceptional exposure compared to sibling controls; no significant differences for other genes
			chr8:67253246–67253686	*CRH*	4		
		Sibling controls N = 60, ♀ = 32, mean age 57	chr16:52383225–52383575	*FTO*	6		
			chr20:56896823–56897145	*GNASA/B*	15		
			chr20:56859210–56859503	*GNASAS*	17		
			chr7:50818080–50818483	*GRB10*	7		
		Group 2	chr6:160346346–160346595	*IGF2R*	10		Group 2
		Prenatal exposure 3rd trim N = 62, ♀ = 34, mean age 59	chr1:205012634–205012962	*IL-10*	4		Hypomethylation of GNASAS*** after exposure during 3rd trim compared to sibling controls; no significant differences for other genes
			chr11:2138912–2139216	*INSIGF*	4		
		Sibling controls N = 62, ♀ = 34, mean age 57	chr11:2677737–2678040	*KCNQ1OT1*	17		
			chr7:127668290–127668646	*LEP*	9		
			chr14:100361166–100361395	*MEG3*	9		
			chr5:142763741–142764104	*NR3C1*	17		
			chr6:2790712–2791113	*TNF*	7		
Heijmans et al. [29]^d^	Dutch Famine*^b^	Group 1	chr11:2126035–2126372	*IGF2*	5	EpiTYPER	Group 1
		Periconceptional exposure N = 60, ♀ = 32, mean age 58					Hypomethylation of *IGF2* CpG 1***, 2–3** and 5** after periconceptional exposure compared to sibling controls
		Sibling controls N = 60, ♀ = 32, mean age 57					
		Group 2					Group 2
		Prenatal exposure 3rd trim N = 62, ♀ = 34, mean age 59					No significant differences for *IGF2*
		Sibling controls N = 62, ♀ = 34, mean age 57					

*^a^Chinese Famine: 1959–1961; *^b^Dutch Famine: 1944–1945; *^c^Bangladesh Famine: 1974–1975; ^d^sample overlap between [72, 74], and between [29, 61, 66, 70];* n.s.* not stated; *ABCA1* ATP-binding cassette subfamily A member 1, *AGTR1* Angiotensin II Receptor Type 1, *AKAP12* A-kinase anchoring protein 12, *APOC1* Apolipoprotein C1, *ATP5B* ATP synthase subunit beta, *BOLA* bolA family member, *CDH23* Cadherin-related 23, *CPT1A* Carnitine palmitoyltransferase 1A, *CRH* Corticotropin-releasing hormone, *CTCF* CCCTC-Binding Factor, *DMR* differentially methylated region, *EXD3* Exonuclease 3′-5′ domain-containing 3, *FTO* Alpha-ketoglutarate-dependent dioxygenase, *GR 1-C* Glucocorticoid receptor, *GNASA/B* G protein alpha S, *GNASAS* GNAS antisense RNA, *GRB10* Growth factor receptor-bound protein 10, *HLA-DQB2* Histocompatibility complex Class 2 DQ Beta 2, *IGF2* Insulin-like growth factor 2, *IGF2R* Insulin-like growth factor 2 receptor, *IL-10* Interleukin-10, *INSIGF* Insulin-induced gene, *INSR* Insulin receptor, *KCNQ1OT1* KCNQ1 opposite strand/antisense transcript 1, *KLF13* Kruppel-like factor 13, *LEP* Leptin, *LPL* Lipoprotein lipase, *LRRC14B* Leucine rich repeat containing 14B, *MEG3* Maternally Expressed 3, *NR3C1* Nuclear receptor subfamily 3 group C member 1, *PARD6G* Par-6 family cell polarity regulator gamma, *PAX8* Paired box 8, *PCR* polymerase chain reaction, *PI3kinase* Phosphatidylinositol 3-kinase p85, *PLD6* Phospholipase D family member 6, *PPARy* Peroxisome proliferator-activated receptor gamma, *PRDM9* PR/SET domain 9, *PRKCA* Protein Kinase C Alpha, *RBM46* RNA-binding motif protein 46, *RFTN1* Raftlin lipid raft linker 1, *SMAD7* SMAD family member 7, *SPG20* Spartin gene, *TNF* Tumor necrosis factor, *VTRNA2-1* Vault RNA 2–1, *ZFP57* Zinc-finger transcription factor 57, *ZFYVE28* Zinc finger FYVE-type containing 28, *ZNF678* Zinc-finger protein 678; *p ≤ 0.05, **p ≤ 0.01, ***p ≤ 0.001

**Table 4:** Effects of prenatal exposure to famine on genome-wide DNA methylation and mental disorders

References	Cohort	Sample description of groups with prenatal or non-exposure	Assessment of symptoms of sychopathology	Chromosome	Gene	No CpG	DNA methylation analysis from blood	Main results
Boks et al. [75]	Chinese Famine*^a^	Prenatally exposed controls N = 25, ♀ = 15, mean age 50	Non-standardized diagnosis accordingto DSM IV criteria	chr6: 291687–293285	*DUSP22*	10	Infinium HumanMethylation 450BeadChip	Hypermethylation of DUSP22** in prenatal exposed SZ patients compared to all other groups
		Prenatally exposed SZ patients N = 23, ♀ = 5, mean age 50						
		Non-exposed controls N = 54, ♀ = 33, mean age 47						
		Non-exposed SZ patients N = 51, ♀ = 23, mean age 47						

*^a^Chinese Famine: 1959–1961; *DUSP22* Dual Specificity Phosphatase 22; *DSM* diagnostic and statistical manual of mental disorders, *SZ* schizophrenia; **p ≤ 0.01

### Risk of bias assessment

The risk of bias assessment is presented in Additional file 2: Table S4. Quality ratings ranged from poor to good, with only two studies rated good on all study items [35, 36].

Of the studies examining symptoms of psychopathology and mental disorders, most scored highest on the statistical methods item. Most studies (86%) used proper statistical analyses and conducted sensitivity analyses. The sample size item was generally rated as good for the mental disorders or symptoms studies (77%). Of the 22 studies, 14 studies (64%) defined famine exposure both quantitatively and qualitatively. Half of the studies (50%) used a good outcome assessment by a psychiatrist or clinical psychologist according to International Classification of Diseases (ICD) or Diagnostic and Statistical Manual of Mental Disorders (DSM) criteria. Only 36% of the studies adjusted for confounders and explained why they did so. 32% of the studies had good sampling representativeness. Sampling representativeness was rated as fair if the sample was drawn from only one hospital registry or survey. The lowest ratings were achieved for the item famine severity assessment, with 55% of the studies failing to include excess death rates (EDR), cohort size shrinkage index (CSSI) or global hunger index (GHI) to measure the severity of famine (for more information, see [37]).

Of the DNA methylation studies, most (73%) used proper statistical analyses and conducted sensitivity analyses. Adjustment for confounding factors was good in 53% of these studies. Only 27% defined famine exposure both quantitatively and qualitatively, and only 27% used a good description of the DNA methylation assay. A small proportion of the studies (13%) had good sampling representativeness and sample size. None of the DNA methylation studies were rated as showing a good famine severity assessment (0%).

### Effects of prenatal famine exposure on offspring symptoms/mental disorders

Twenty-two studies investigated the effect of prenatal famine exposure on offspring symptoms of psychopathology and/or mental disorders.

As shown in Table 1, one study found higher psychopathology, as measured with the Mental Health Inventory (MHI-5) in individuals who experienced famine during prenatal development compared to individuals who did not [38]. Five studies reported increased depressive symptoms [39–43] in individuals with prenatal famine exposure compared to individuals with non-prenatal exposure and/or non-exposure. One study reported an association between prenatal exposure to famine and increased anxiety and depressive symptoms, as measured with the HADS [44]. In contrast, another study found no significant association between prenatal famine exposure and anxiety and depressive symptoms (HADS) as compared to non-prenatal exposure and non-exposure [45].

With regard to mental disorders, one study found a generally increased risk of mental disorders [46] after prenatal exposure compared to non-exposure. Six studies consistently reported an increased risk of schizophrenia after prenatal exposure compared to non-prenatal and/or non-exposure to famine [35, 36, 47–50]. In contrast, one study found a higher risk of developing schizophrenia in adults with non-exposure to famine than in adults with prenatal exposure [51]. An increased risk of major affective disorders was found to be linked to in utero exposure to famine as compared to non-exposure in two studies [52, 53]. One study reported an increased risk of antisocial personality disorder [54] and another an increased risk of schizoid personality disorder [55] in men after prenatal exposure compared to non-exposure to famine. Addictive disorders [56] and addictive behaviors [57] in adults were related to prenatal famine exposure but not to non-prenatal famine exposure.

**Fig. 2:**
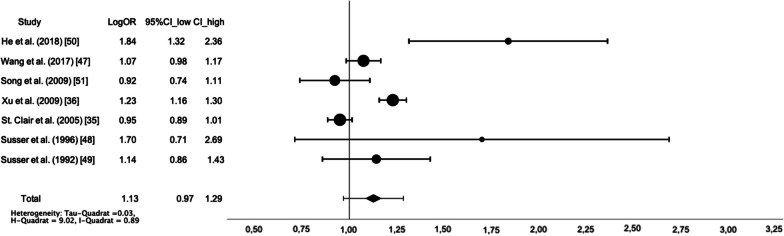
Forest plot of studies comparing adults prenatally exposed to famine with adults non-prenatally and non-exposed to famine regarding risk of developing schizophrenia. Conducting subgroup analyses for the Dutch and the Chinese famine did not alter the results

In terms of depressive symptoms, two studies [39, 42] provided sufficient data for meta-analysis based on OR, with results varying by exposure period. On the one hand, adults prenatally exposed to famine showed a decreased risk of depressive symptoms compared to adults with no exposure to famine and adults who were exposed to famine after gestation (logOR = 0.96, 95% CI [0.79, 1.14]; Z = 10.75, *p* < 0.001; Q = 8.56, I^2^ = 88%). On the other hand, adults prenatally exposed to famine showed an increased risk of depressive symptoms compared to adults with no exposure to famine (logOR = 1.14, 95% CI [0.94, 1.34]; Z = 11.31, *p* < 0.001; Q = 6.87, I^2^ = 86%). In terms of anxiety and depressive symptoms as measured by the HADS, meta-analysis confirmed the null-findings (HADS-A: g = 0.08, 95% CI [− 0.05, 0.21]; Z = 1.17, *p* = 0.241; Q = 0, I^2^ = 0%; HADS-D: g = 0.06, 95% CI [− 0.08, 0.19]; Z = 0.84, *p* = 0.403; Q = 0.23, I^2^ = 0%). Meta-analysis confirmed the increased risk of suffering from schizophrenia in adulthood after prenatal famine exposure compared to non-prenatal exposure and non-exposure together (logOR = 1.13, 95% CI [0.97, 1.29]; Z = 13.97, *p* < 0.001). Heterogeneity was high (Q = 9.02, I^2^ = 89%), see Fig. 2. The results remained unchanged when subgroup analyses were conducted for the Dutch and the Chinese famine (two Dutch famine studies: logOR = 1.21, 95% CI [0.85, 1.57]; Z = 6.57, *p* < 0.001; Q = 1.13, I^2^ = 11% and five Chinese famine studies: logOR = 1.12, 95% CI [0.92, 1.33]; Z = 10.74, *p* < 0.001; Q = 18.25, I^2^ = 95%). Insufficient data were available for meta-analyses on major affective disorders, antisocial and schizoid personality disorder, as well as addictive disorders.

### Effects of prenatal famine exposure on offspring DNA methylation (epigenome-wide analysis)

Nine studies, which are listed in Table 2, investigated DNA methylation by conducting (epi)genome-wide analysis in adults prenatally exposed to famine [58–66]. All of these used whole blood as tissue.

Four studies determined DNA methylation using the HumanMethylation450 BeadChip microarray, which has a coverage of over 450,000 sites [67, 68]. The first of these four studies did not find significantly differentially methylated regions (DMRs) in adult offspring following prenatal famine exposure as compared to non-prenatal exposure and non-exposure [59]. The second study identified that prenatal exposure to famine during early gestation was significantly associated with 613 DMRs as compared to non-exposure [58]. The authors specifically reported hypomethylated regions in four genes, namely *CCDC51*, *TMA7*, *ENO2* and *ZNF226* [58]. The third study found a variety of hyper- (*FAM150B/TMEM18*, *PPAP2C*, *SLC38A2)* and hypomethylated (*OSBPL5/MRGPRG)* genes in adult offspring exposed to famine during early gestation as compared to time and sibling controls. In addition, exposure during conception was associated with decreased methylation of *TMEM105/SLC38A10*, and exposure during any week of gestation was associated with increased methylation of the genes *TACC1* and *ZNF385A* compared to time and sibling controls [60].

Lastly, an association was found between prenatal famine exposure and hypo-methylation of the genes *CRELD2*, *LRRC8D*, *LOC100132354*, *OSBPL5*/*MRGPRG*, *TXNIP*, *PFKFB3* as well as hypermethylation of the genes *ABCG1*, *CCDC155*, *FAM150B*, *METTL8*, *PNPO*, *PPAP2C*, *SLC38A2*, *SYNGR1*, *TACC1* and *ZNF385A* compared to controls [64].

Two studies used methylation analyses, which cover over 850,000 sites [69]. One study reported evidence of 601 hypermethylated and 360 hypomethylated sites after prenatal famine exposure as compared to time controls [63]. The other study reported no significant differentially methylated sites after controlling for multiple testing [65].

The two studies measuring global DNA methylation via pyrosequencing did not find a link between prenatal famine exposure and altered methylation patterns as compared to sibling controls and time controls [62, 66]. One of these studies also analyzed global DNA methylation via MethyLight and LUminometric Methylation Assay (LUMA), yielding no significant findings [62].

One study used reduced representation bisulfite sequencing (RRBS) to assess DMRs and found hypermethylation in 60.8% out of 181 identified sites and hypomethylation in 39.2% following periconceptional exposure to famine compared to sibling controls [61]. In the present analysis, we solely reported on genes for which there was a significant association between DNA methylation and prenatal famine exposure. Using the data published in the included papers, we verified whether genes that were significant in some studies were also significant in others, and mostly found no concordance. For instance, only six genes identified by Tobi et al. [60] were replicated in another study by Tobi et al. [64], even though methylation analysis was performed on the same sample. Meta-analysis was not suitable due to different DNA methylation microarrays/data processing approaches and partially unavailable data.

### Effects of prenatal famine exposure on offspring DNA methylation (candidate gene analysis)

As can be seen in Table 3, candidate gene DNA methylation analyses revealed significant associations between prenatal famine exposure and a variety of hyper- and hypomethylated genes as compared to the different control groups.

Compared to sibling controls, periconceptional famine exposure was associated with hypomethylation of *KLF13* [61], *IGF2* [29, 66], and *INSIGF* [66, 70]. Besides periconceptional exposure, prenatal exposure during late gestation was associated with hypomethylation of the *GNASAS* gene [70]. Compared to sibling and time controls, prenatal exposure to famine was related to hypermethylation in several genes (*CDH23, CPT1A, INSR, SMAD7* [61]; *ABCA1, IL-10, LEP, GNASAS* and *MEG* [70]). Compared to time controls only, prenatal famine exposure was related to hypomethylation of the *AGTR1* and *PRKCA* genes [63] and hypermethylation of the *IGF2* and *INSR* genes [72].

As compared to non-prenatal exposure and non-exposure, adults prenatally exposed to famine showed decreased methylation of the *ZFP57* and *PRDM9* genes and increased methylation of the *PAX8* gene [59]. Moreover, prenatal exposure to famine was related to hypomethylation of *VTRNA2-1* and *EXD3* compared to non-prenatal exposure only [59]. One study reported no association of *GR 1-C, LPL*, *PI3kinase*, and *PPARy* with in utero exposure to famine compared to non-prenatal exposure and non-exposure [73].

In sum, the candidate genes most affected by prenatal famine exposure are *IGF2* and *INSR*. In addition, prenatal famine exposure was not associated with several other candidate genes, which are reported in Table 3 [59, 61, 70, 73, 74].

Although a few significant candidate genes were replicated in other studies, it is possible that methylation analyses were performed on the same sample. Candidate-gene studies were not eligible for meta-analysis due to the heterogeneity of affected genes and partially unavailable data.

### DNA methylation as a mediator between famine exposure during pregnancy and mental disorders

Table 4 presents a more recent study by Boks et al. [75], who analyzed changes in DNA methylation in individuals exposed to famine during the first 3 months of prenatal development and their susceptibility to schizophrenia in adulthood. The authors reported that prenatally exposed adults with schizophrenia showed hypermethylation of the *DUSP22* gene compared to non-exposed patients and healthy controls [75].

## Discussion

In the present systematic review and meta-analysis, we investigated the association between prenatal famine exposure, DNA methylation and mental disorders in adult offspring. We report three main findings: First, meta-analysis confirmed that exposure to famine during prenatal development increases the offspring’s risk of suffering from schizophrenia. With regard to depression, meta-analyses yielded contradictory findings, showing either increased or decreased risk of depressive symptoms depending on exposure periods. Anxiety and depressive symptoms, as measured with the HADS, were not associated with prenatal famine exposure. Prenatal famine exposure was further associated with addictive disorders and behaviors as well as antisocial and schizoid personality disorder. Second, we found that prenatal famine exposure is associated with hypo- and hypermethylation of a variety of genes. The largest number of studies reported differences in DNA methylation of the *IGF2* gene. Third, only one mediation study has been conducted to date, which described altered DNA methylation of the *DUSP22* gene as a potential mechanism underlying the association between prenatal famine exposure and schizophrenia in adult offspring.

With regard to the first finding, additional studies confirm the increased risk for the development of schizophrenia in offspring prenatally exposed to a (natural) disaster such as an earthquake [76, 77], a terrorist attack [78], infections, and lead exposure [79]. There are several potential reasons for this effect of unfavorable environmental circumstances on an increased susceptibility to schizophrenia. According to the neurodevelopmental hypothesis proposed by Weinberger [80] and Murray and Lewis [81], such conditions impair the neurodevelopment of the fetus by adversely altering gene expression [81–87]. In particular, shortly after fertilization, a complete demethylation of the genome occurs, which is then re-established during embryogenesis [88]. Adverse environmental circumstances during this periconceptional period can thus permanently alter the DNA methylation of genes involved in neural pathways, impair brain development, and predispose the offspring to an increased risk of schizophrenia [84]. Moreover, researchers have found that schizophrenia shares common features with other mental disorders such as schizoaffective disorders and depression [89, 90], suggesting that the same epigenetic mechanisms are involved in its pathogenesis. However, the inconclusive findings of the meta-analyses on depressive symptoms may also be explained by the fact that environmental conditions influence DNA methylation at other life stages, in addition to early prenatal development [91]. Indeed, offspring exposed to famine in infancy or childhood exhibit more depressive symptoms than offspring exposed to famine prenatally. Nevertheless, prenatal exposure to famine increases the risk of depressive symptoms in adult offspring compared to offspring who have never been exposed to famine. Furthermore, the inconclusive findings regarding depressive symptoms and the null findings regarding anxiety may be attributable to the fact that only two studies could be included in the meta-analyses due to the heterogeneity of the examined exposure periods and different methods of statistical analysis.

With respect to the finding that *IGF2* appears to be the gene that is most affected by prenatal famine exposure, the studies in this review revealed both hyper- and hypomethylation of the *IGF2* gene in offspring. The reason for this finding of both increased and decreased methylation, despite the fact that all offspring were prenatally exposed to famine, might lie in a dose–response relationship in terms of duration and severity of prenatal famine exposure and *IGF2* DNA methylation. Specifically, the Chinese famine was more severe and lasted for longer (3 years) compared to the Dutch famine, which was less severe and lasted for only 6 months [92]. More severe and longer exposure may have led to increased DNA methylation [72], whereas shorter and less severe exposure may have resulted mainly in decreased methylation of the *IGF2* gene [29, 66]. This assumption is in line with the study by Shen et al. [92], who reported increased methylation of the *IGF2* gene in offspring exposed to severe famine compared to offspring exposed to moderate famine. Moreover, different genomic positions annotated to the *IGF2* gene were examined [29, 66], which could be another reason for differences in the direction of DNA methylation.

As for the third finding, there is evidence that *DUSP* family genes are involved in neural functions and play a role in the pathophysiology of mental disorders such as depression, bipolar disorder, and schizophrenia [93]. This supports the involvement of the *DUSP22* gene in the etiology of schizophrenia in adults prenatally exposed to famine [75]. In addition, we suggest that altered DNA methylation of the aforementioned *IGF2* gene may contribute to an increased risk of mental disorders, as this gene is also involved in neuronal functions. Specifically, it is an important contributor to fetal growth and development of the central nervous system [94–96], with increased methylation of the *IGF2* gene in the placenta, for example, showing an association with higher birth weight [94]. However, another study found that increased methylation of this gene (in maternal blood) was associated with lower birth weight [97], and others found no significant association [98]. In terms of the central nervous system, dysregulations of this gene are associated with various mental disorders such as depression and schizophrenia [99].

The phenotype of adults prenatally exposed to famine may additionally be caused by altered DNA methylation of candidate genes in the neuroendocrine and immune systems [17, 100, 101]. Specifically, the *LEP* gene affects the HPA axis activity by inhibiting the release of corticotropin-releasing hormone (CRH), thereby suppressing its activity and reducing glucocorticoid production [102–104]. Hypermethylation of the *LEP* gene can lead to decreased gene expression [105] and possibly inhibits its role in suppressing HPA axis activity. In addition, hypermethylation of this gene has been associated with schizophrenia [106], and hyperactivity of the HPA axis is an underlying biological mechanism of depression [107, 108]. The findings of our review demonstrate that prenatal famine exposure is associated with hypermethylation of the *LEP* gene in adult offspring [70]. Furthermore, the function of the neuroendocrine system is closely linked to the function of the immune system, and the HPA axis acts as a mediator between the two systems [109–112]. The *IL-10* gene, an anti-inflammatory cytokine of the immune system, influences the HPA axis activity [112–114] by increasing the production of CRH and adrenocorticotropic hormone (ACTH) in the pituitary [109, 110]. Differences in its gene expression have been found in adults suffering from a major affective disorder or schizophrenia [115–117]. Evidence indicates that prenatal exposure to famine is related to increased methylation of the *IL-10* gene in adult offspring [70].

The present review is the first to systematically and quantitatively present the effects of prenatal famine exposure on both mental disorders or symptoms of psychopathology and DNA methylation. Its strengths include the comprehensive literature search and rigorous quality assessment (risk of bias). However, the results of the meta-analyses, particularly the omission of a meta-analysis for the whole-genome DNA methylation results, should be interpreted with caution because the authors did not to obtain all affected genes from all whole-genome DNA methylation analysis studies. In addition, we are unable to rule out publication bias due to the very small number of studies suitable for meta-analyses. All methylation studies presented in this review used whole blood as a tissue. One might consider whether DNA methylation in peripheral specimens serves as a marker for DNA methylation in brain tissue as there is evidence that epigenetic differences in peripheral specimens do not always correlate with differences in brain tissue [118, 119]. For example, Walton et al. [120] found that only 7.9% of CpGs were broadly correlated between blood and living brain tissue from the same individuals. However, they were able to identify CpG markers from blood tissue that significantly correlated with brain tissue and were involved in biological pathways affected in individuals with schizophrenia [120]. As a further limitation, the heterogeneity of genes affected by prenatal famine exposure might result from the lack of power of small sample sizes and different DNA methylation techniques across the included studies. However, it is noteworthy that most of the associations found were statistically significant at the *p* < 0.001 level (Tables 2, 3 and 4), even after Bonferroni correction [65, 70, 72, 74] and Benjamin-Hochberg adjustment [60, 66] for multiple testing. Candidate gene analyses have the distinct advantage of enabling a more thorough investigation of specific regions of interest by assessing the overall methylation of a target region and allowing researchers to identify specific CpG sites involved in disease pathogenesis [121]. Epigenome-wide DNA methylation analyses enable the analysis of the entire genome, as generally speaking, more than one gene is involved in the pathogenesis of diseases [122], but cover only small numbers of CpG sites per gene [123, 124]. Moreover, as the examined famine cohorts were geographically diverse, the different methylated genes may be attributable to ethnicity. For instance, Elliott et al. [125] found large differences in DNA methylation between European and South Asian individuals due to ethnically different cell composition. Additionally, the cause of the famines also differed, with the Dutch famine being the result of a food embargo during World War II [23] and the Chinese famine being due to political and economic mismanagement combined with drought [126]. This may further have exposed the two cohorts to distinct psychosocial stressors, which might have influenced their DNA methylation differently.

## Conclusion

Prenatal famine exposure has been associated with altered DNA methylation of genes involved in neuronal, neuroendocrine, and immune processes, which may causally promote the development of mental disorders, specifically schizophrenia and depression in adult offspring. Further genome-wide and hypothesis-driven candidate gene mediation analyses, preferably with a longitudinal design and large sample sizes, are warranted to obtain a complete picture of the role of DNA methylation in the association between prenatal exposure to famine and the development of mental disorders. A better understanding may improve the diagnosis and treatment of schizophrenia and depression, as DNA methylation can be reversed by pharmacological drugs [127–129], and may inform the development of nutritional intervention programs for pregnant women affected by famine.

### Supplementary Information

**Additional file 1: Table S1.** Quality assessment scale (risk of bias) of adults prenatally exposed to famine who suffered from symptoms of psychopathology or a mental disorder; modified from Li and Lumey [31] and Newcastle–Ottawa Scale by Wells et al. [32]. **Table S2.** Quality assessment scale (risk of bias) of adults prenatally exposed to famine with alterations in (epi)genome-wide DNA methylation; modified from Li and Lumey [31] and Newcastle–Ottawa Scale by Wells et al. [32]. **Table S3.** Quality assessment scale (risk of bias) of adults prenatally exposed to famine with alterations in candidate gene DNA methylation; modified from Li and Lumey [31] and Newcastle–Ottawa Scale by Wells et al. [32].

**Additional file 2: Table S4.** Risk of bias assessment for the effect of famine on symptoms of psychopathology/mental disorders, and DNA methylation.

## Data Availability

The datasets used and/or analyzed during the current study are available from the corresponding author on reasonable request.

## Abbreviations

ABCA1: ATP-binding cassette subfamily A member 1
ABCG1: ATP binding cassette subfamily G member 1
ACTH: Adrenocorticotropic hormone
ADHD: Attention deficit hyperactivity disorder
AGTR1: Angiotensin II receptor type 1
AKAP12: A-kinase anchoring protein 12
APOC1: Apolipoprotein C1
ASPD: Antisocial personality disorder
ATP5B: ATP synthase subunit beta
BOLA: Bola family member
CCDC51: Coiled-coil domain containing 51
CCMD: Chinese classification of mental disorders
CDH23: Cadherin-related 23
CES-D: Center for epidemiologic studies depression scale
CpG: Cytosine guanine dinucleotides
CPT1A: Carnitine palmitoyltransferase 1A
CRELD2: Cysteine rich with EGF-like domains 2
CRH: Corticotropin-releasing hormone
CSSI: Cohort size shrinkage index
CTCF: CCCTC-binding factor
DMR: Differentially methylated region
DNA: Deoxyribonucleic acid
DUSP22: Dual specificity phosphatase 22
DSM: Diagnostic and statistical manual of mental disorders
EDR: Excess death rate
ENO2: Enolase 2
EXD3: Exonuclease 3′–5′ domain containing 3
FTO: Alpha-ketoglutarate-dependent dioxygenase
FAM150B: Family with sequence similarity 150 member B
GDS: Geriatric depression scale
GHI: Global hunger index
GHQ-12: General health questionnaire
GNASA/B: G protein alpha S
GNASAS: GNAS antisense RNA
GRB10: Growth factor receptor-bound protein 10
GR 1-C: Glucocorticoid receptor
HADS-A/-D: Hospital anxiety and depression scale
HLA-DQB2: Histocompatibility complex class II DQ beta 2
HPA: Hypothalamic–pituitary–adrenal
ICD-10: International statistical classification of diseases and related health problems
IGF2R: Insulin-like growth factor 2 receptor
IGF2: Insulin-like growth factor 2
INSIGF: Insulin-induced gene
INSR: Insulin receptor
KCNQ1OT1: KCNQ1 opposite strand/antisense transcript 1
KLF13: Kruppel-like factor 13
LEP: Leptin
LINE-1: Long interspersed nucleotide element-1
LOC10012354: LOC100132354
LPL: Lipoprotein lipase
LRRC8D: Leucine rich repeat containing 8 VRAC subunit D
LRRC14B: Leucine rich repeat containing 14B
LUMA: Luminometric methylation assay
MEG3: Maternally expressed 3
MHI-5: Mental health inventory
MRGPRG: MAS related GPR family member G
NR3C1: Nuclear receptor subfamily 3 group C member 1
OSBPL5: Oxysterol binding protein-like 5
OSF: Open science framework
OR: Odds ratio
PARD6G: Par-6 family cell polarity regulator gamma
PAX8: Paired box 8
PCR: Polymerase chain reaction
PI3kinase: Phosphatidylinositol 3-kinase p85
PLD6: Phospholipase D family member 6
PNPO: Pyridoxamine 5′-phosphate oxidase
PPAP2C: Phosphatidic acid phosphatase 2c
PPARy: Peroxisome proliferator-activated receptor gamma
PRDM9: PR/SET domain 9
PRISMA-P: Preferred reporting items for systematic review and meta-analysis protocols
PRKCA: Protein kinase C alpha
RBM46: RNA-binding motif protein 46
RFTN1: Raftlin lipid raft linker 1
RRBS: Reduced representation bisulfite sequencing
Sat2: Satellite repeat-2
SLC28A2: Solute carrier family 38 member 2
SLC38A10: Solute carrier family 38 member 10
SMAD7: SMAD family member 7
SPG20: Spartin gene
SYNGR1: Synaptogyrin 1
SZ: Schizophrenia
TACC1: Transforming acidic coiled-coil-containing protein 1
TMA7: Translation machinery-associated protein 7
TMEM18: Transmembrane protein 18
TMEM105: TMEM105 long non-coding RNA
TNF: Tumor necrosis factor
TXNIP: Thioredoxin interacting protein
VTRNA2-1: Vault RNA 2-1
ZFP57: Zinc-finger transcription factor 57
ZFYVE28: Zinc-finger FYVE-type containing 28
ZNF226: Zinc-finger protein 226
ZNF385A: Zinc-finger protein 385A
ZNF678: Zinc-finger protein 678
