# Supplementary material for: The association between prenatal famine, DNA methylation and mental disorders: a systematic review and meta-analysis
Source: Clin Epigenetics. 2023 Sep 16;15:152. doi: 10.1186/s13148-023-01557-y (PMC10505322; doi:10.1186/s13148-023-01557-y)
Supplement: Supplementary file 1 — Additional file 1: Table S1. Quality assessment scale (risk of bias) of adults prenatally exposed to famine who suffered from symptoms of psychopathology or a mental disorder; modified from Li and Lumey [31] and Newcastle–Ottawa Scale by Wells et al. [32]. Table S2. Quality assessment scale (risk of bias) of adults prenatally exposed to famine with alterations in (epi)genome-wide DNA methylation; modified from Li and Lumey [31] and Newcastle–Ottawa Scale by Wells et al. [32]. Table S3. Quality assessment scale (risk of bias) of adults prenatally exposed to famine with alterations in candidate gene DNA methylation; modified from Li and Lumey [31] and Newcastle–Ottawa Scale by Wells et al. [32]. [file 13148_2023_1557_MOESM1_ESM.doc]

**Additional file 1: Table S1.** Quality assessment scale (risk of bias) of adults prenatally exposed to famine who suffered from symptoms of psychopathology or a mental disorder; modified from Li and Lumey [31] and Newcastle-Ottawa Scale by Wells et al. [32]

1. Sampling representativeness

Good: the study population can reflect the disease pattern among individuals with and without famine exposure specifically and properly

Fair: the study population has the potential to reflect the disease pattern among individuals with and without famine exposure

Poor: the study population cannot reflect the disease pattern among individuals with and without famine exposure

1. Sample size

Good: the monthly number of exposed subjects is over 40

Fair: the monthly number of exposed subjects is between 10 and 40

Poor: the monthly number of exposed subjects is below 10 or not reported or cannot be calculated

1. Exposure definition

Good: the exposure is defined both quantitatively and qualitatively

Fair: the exposure is defined either quantitatively or qualitatively

Poor: the exposure definition is not justified quantitatively or qualitatively

1. Famine severity assessment

Good: different data sources are used to evaluate famine severity

Fair: single data source is used to evaluate famine severity

Poor: no data is used or reported to evaluate famine severity

1. Confounding adjustment

Good: the confounding adjustment is conducted with explanation or discussion

Fair: the confounding adjustment is conducted without explanation or discussion

Poor: the confounding adjustment is not conducted or reported

1. Outcome assessment – Mental disorders

Good: the outcome is assessed according to ICD or DSM by a psychiatrist or clinical psychologist

Fair: the outcome is assessed according to ICD or DSM by a trained student or self-report

Poor: the outcome assessment method is not described

1. Statistical methods

Good: the proper statistical analysis is conducted and supplemented by sensitivity analysis

Fair: the proper statistical analysis is conducted

Poor: the statistical analysis is not properly conducted or clearly reported

**Additional file 1: Table S2.** Quality assessment scale (risk of bias) of adults prenatally exposed to famine with alterations in (epi)genome-wide DNA methylation; modified from Li and Lumey [31] and Newcastle-Ottawa Scale by Wells et al. [32]

1) Sampling representativeness

Good: the study population can reflect the disease pattern among individuals with and without famine exposure specifically and properly

Fair: the study population has the potential to reflect the disease pattern among individuals with and without famine exposure

Poor: the study population cannot reflect the disease pattern among individuals with and without famine exposure

2) Sample size

Good: the sample size is 80 or above for each group (exposed, non-exposed and non-prenatally exposed)

Fair: the sample size is 50 or above in at least one group (exposed, non-exposed and non-prenatally exposed)

Poor: the sample size is under 50 for each group (exposed, non-exposed and non-prenatally exposed)

3) Exposure definition

Good: the exposure is defined both quantitatively and qualitatively

Fair: the exposure is defined either quantitatively or qualitatively

Poor: the exposure definition is not justified quantitatively or qualitatively

4) Famine severity assessment

Good: different data sources are used to evaluate famine severity

Fair: single data source is used to evaluate famine severity

Poor: no data is used or reported to evaluate famine severity

5) Confounding adjustment

Good: the confounding adjustment is conducted with explanation or discussion

Fair: the confounding adjustment is conducted without explanation or discussion

Poor: the confounding adjustment is not conducted or reported

6) Outcome assessment – DNA methylation

Good: DNA methylation assay is well described and 3-5 of the criteria a) standardization regarding time of day, b) storage at a maximum of -80°C, c) adequate removal of samples, d) definition of Δβ minimum, and e) FDR≤.05 are stated.

Fair: DNA methylation assay is described and 1-2 of the criteria a) standardization regarding time of day, b) storage at a maximum of -80°C, c) adequate removal of samples, d) definition of Δβ minimum, and e) FDR≤.05 are stated.

Poor: DNA methylation assay is described and none of the criteria a) standardization regarding time of day, b) storage at a maximum of -80°C, c) adequate removal of samples, d) definition of Δβ minimum, and e) FDR≤.05 are stated.

7) Statistical methods

Good: the proper statistical analysis is conducted and supplemented by sensitivity analysis

Fair: the proper statistical analysis is conducted

Poor: the statistical analysis is not properly conducted or clearly reported

**Additional file 1: Table S3.** Quality assessment scale (risk of bias) of adults prenatally exposed to famine with alterations in candidate gene DNA methylation; modified from Li and Lumey [31] and Newcastle-Ottawa Scale by Wells et al. [32]

1) Sampling representativeness

Good: the study population can reflect the disease pattern among individuals with and without famine exposure specifically and properly

Fair: the study population has the potential to reflect the disease pattern among individuals with and without famine exposure

Poor: the study population cannot reflect the disease pattern among individuals with and without famine exposure

2) Sample size

Good: the sample size is 80 or above for each group (exposed, non-exposed and non-prenatally exposed)

Fair: the sample size is 50 or above in at least one group (exposed, non-exposed and non-prenatally exposed)

Poor: the sample size is under 50 for each group (exposed, non-exposed and non-prenatally exposed)

3) Exposure definition

Good: the exposure is defined both quantitatively and qualitatively

Fair: the exposure is defined either quantitatively or qualitatively

Poor: the exposure definition is not justified quantitatively or qualitatively

4) Famine severity assessment

Good: different data sources are used to evaluate famine severity

Fair: single data source is used to evaluate famine severity

Poor: no data is used or reported to evaluate famine severity

5) Confounding adjustment

Good: the confounding adjustment is conducted with explanation or discussion

Fair: the confounding adjustment is conducted without explanation or discussion

Poor: the confounding adjustment is not conducted or reported

6) Outcome assessment – Candidate gene analyses

Good: DNA methylation assay is well described and 3-4 of the criteria a) standardized regarding time of day, b) storage at a maximum of -70°C, c) CpG success rate >75% and d) replicate SD <5% are stated.

Fair: DNA methylation assay is described and 1-2 criteria a) standardized regarding time of day, b) storage at a maximum of -70°C, c) CpG success rate >75% and d) replicate SD <5% are stated.

Poor: DNA methylation assay is mentioned but none of the criteria a) standardized regarding time of day, b) storage at a maximum of -70°C, c) CpG success rate >75% and d) replicate SD <5% are stated.

7) Statistical methods

Good: the proper statistical analysis is conducted and supplemented by sensitivity analysis

Fair: the proper statistical analysis is conducted

Poor: the statistical analysis is not properly conducted or clearly reported
